# 1-Methyl-2,4-bis­(2-methoxy­phen­yl)-3-aza­bicyclo­[3.3.1]nonan-9-one

**DOI:** 10.1107/S1600536809047928

**Published:** 2009-11-18

**Authors:** P. Parthiban, V. Ramkumar, Yeon Tae Jeong

**Affiliations:** a Division of Image Science and Information Engineering, Pukyong National University, Busan 608 739, Republic of Korea; b Department of Chemistry, IIT Madras, Chennai, TamilNadu, India

## Abstract

The crystal structure of the title compound, C_23_H_27_NO_3_, shows that the compound exists in a chair–chair conformation with an equatorial disposition of 2-methoxy­phenyl groups at an angle of 39.94 (3)° with respect to each other. An inter­molecular N—H⋯π inter­action is observed in the crystal packing.

## Related literature

For the biological activity of 3-aza­bicyclo­nona­nes, see: Barker *et al.* (2005 ▶); Hardick *et al.* (1996 ▶); Jeyaraman & Avila (1981 ▶). For related structures with similar conformations, see: Parthiban *et al.* (2008 ▶); Parthiban, Ramkumar & Jeong (2009 ▶); Parthiban, Ramkumar, Kim *et al.* (2009 ▶). For a related structure with a chair–boat conformation, see: Smith-Verdier *et al.* (1983 ▶). For a related structure with a boat–boat conformation, see: Padegimas & Kovacic (1972 ▶). For ring puckering parameters, see: Cremer & Pople (1975 ▶); Nardelli (1983 ▶).
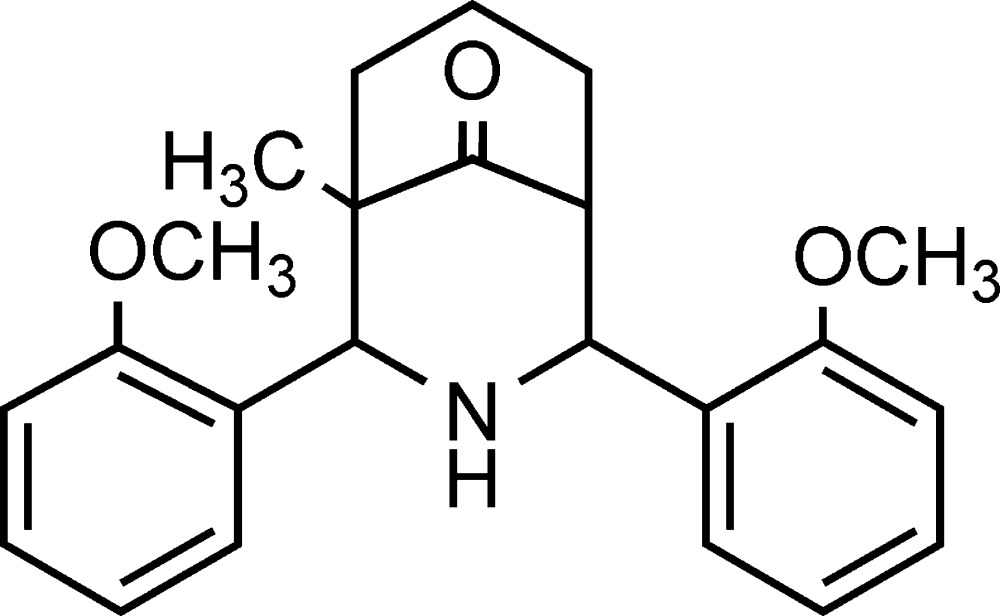


## Experimental

### 

#### Crystal data

C_23_H_27_NO_3_
*M* *_r_* = 365.46
Monoclinic, 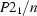

*a* = 7.9569 (3) Å
*b* = 20.8291 (9) Å
*c* = 11.6708 (6) Å
β = 96.297 (2)°
*V* = 1922.59 (15) Å^3^
*Z* = 4
Mo *K*α radiation
μ = 0.08 mm^−1^
*T* = 298 K
0.41 × 0.24 × 0.20 mm

#### Data collection

Bruker APEXII CCD area-detector diffractometer
Absorption correction: multi-scan (*SADABS*; Bruker, 1999 ▶) *T*_min_ = 0.288, *T*_max_ = 0.980
14049 measured reflections
4608 independent reflections
3166 reflections with *I* > 2σ(*I*)
*R*_int_ = 0.026

#### Refinement

*R*[*F* ^2^ > 2σ(*F* ^2^)] = 0.048
*wR*(*F* ^2^) = 0.127
*S* = 1.02
4608 reflections
251 parameters
H atoms treated by a mixture of independent and constrained refinement
Δρ_max_ = 0.22 e Å^−3^
Δρ_min_ = −0.21 e Å^−3^

### 

Data collection: *APEX2* (Bruker, 2004 ▶); cell refinement: *APEX2* and *SAINT-Plus* (Bruker, 2004 ▶); data reduction: *SAINT-Plus* and *XPREP* (Bruker, 2004 ▶); program(s) used to solve structure: *SHELXS97* (Sheldrick, 2008 ▶); program(s) used to refine structure: *SHELXL97* (Sheldrick, 2008 ▶); molecular graphics: *ORTEP-3* (Farrugia, 1997 ▶) and *Mercury* (Macrae *et al.*, 2006 ▶); software used to prepare material for publication: *SHELXL97*.

## Supplementary Material

Crystal structure: contains datablocks global, I. DOI: 10.1107/S1600536809047928/ez2190sup1.cif

Structure factors: contains datablocks I. DOI: 10.1107/S1600536809047928/ez2190Isup2.hkl

Additional supplementary materials:  crystallographic information; 3D view; checkCIF report

## Figures and Tables

**Table 1:** Hydrogen-bond geometry (Å, °)

*D*—H⋯*A*	*D*—H	H⋯*A*	*D*⋯*A*	*D*—H⋯*A*
N1—H1*A*⋯*Cg*1^i^	0.862 (15)	2.852 (3)	3.6276 (14)	150.6 (12)

Symmetry code: (i) 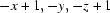. *Cg*1 is the centroid of the C16–C21 ring.

**Fig. 1.**
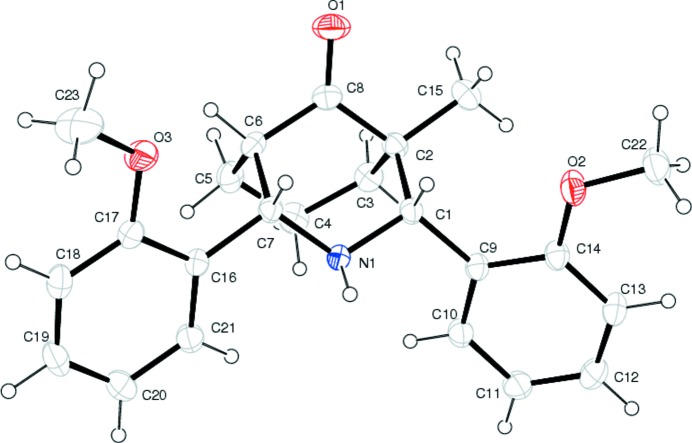
ORTEP diagram of the molecule, showing the atom numbering scheme, with atoms represented as 30% probability ellipsoids.

## supplementary crystallographic information

### Comment

3-Azabicyclononanes are an important class of heterocycles due to their broad spectrum biological activities (Jeyaraman & Avila, 1981; Hardick *et al.*, 1996; Barker *et al.*, 2005). Owing to the diverse possibilities in conformations, *viz*., chair-chair (Parthiban *et al.*, 2008; Parthiban, Ramkumar & Jeong, 2009; Parthiban, Ramkumar, Kim *et al.*, 2009), chair-boat (Smith-Verdier *et al.*, 1983) and boat-boat (Padegimas & Kovacic, 1972) for the azabicycle, the present crystal study was undertaken to explore the conformation, stereochemistry and bonding of the title compound.

The analysis of torsion angles, asymmetry parameters and least-squares planes calculated for the title compound shows that the piperidine ring adopts a near ideal chair conformation with deviations of the ring atoms C8 and N1 from the C1/C2/C6/C7 plane by 0.655 (3) Å and -0.708 (3) Å, respectively. The smallest displacement asymmetry parameters are q2 = 0.0341 (15) Å and q3 =0.6123 (15) Å (Nardelli, 1983). The total puckering amplitude, Q_T_ = 0.6132 (15) Å and θ = 3.14 (14) ° (Cremer & Pople, 1975). The cyclohexane ring deviates from the ideal chair conformation by the deviation of ring atoms C4 and C8 from the C2/C3/C5/C6 plane by -0.697 (4) Å and 0.535 (3) Å, respectively. The smallest displacement asymmetry parameters are q2 = 0.1216 (17) Å and q3 = 0.5322 (17) Å (Nardelli, 1983); total puckering amplitude, Q_T_ = 0.5460 (16) Å, and θ =12.87 (18)° (Cremer & Pople, 1975). Hence, the title compound C_23_H_27_NO_3_, exists in a chair-chair conformation with an equatorial orientation of the *ortho*-methoxyphenyl groups on the heterocycle, which are orientated at an angle of 39.94 (3)° with respect to each other. The crystal structure is stabilized by an intermolecular N-H···π interaction between N1-H1A and the C16/C17/C18/C19/C20/C21 ring in a neighbouring molecule [N···centroid distance of 2.852 (3)Å; symmetry operator: 1-x,-y,1-z].

### Experimental

A mixture of 2-methylcyclohexanone (0.05 mol, 5.61 g) and *ortho*-methoxybenzaldehyde (0.1 mol, 13.62 g) was added to a warm solution of ammonium acetate (0.075 mol, 5.78 g) in 50 ml of absolute ethanol. The mixture was gently warmed with stirring until a yellow color was obtained during the mixing of the reactants and then allowed to stir at 303–308° K until formation of the product. At the end, the crude azabicyclic ketone was separated by filtration and washed with a 1:5 ethanol-ether mixture until the solid became colorless. Recrystallization of the compound from ethanol gave X-ray diffraction quality crystals of 1-methyl-2,4-bis(2-methoxyphenyl)-3- azabicyclo[3.3.1]nonan-9-one.

### Refinement

Nitrogen H atoms were located in a difference Fourier map and refined isotropically. Other hydrogen atoms were fixed geometrically and allowed to ride on the parent carbon atoms,with aromatic C—H =0.93 Å, aliphatic C—H = 0.98Å and methylene C—H = 0.97 Å. The displacement parameters were set for phenyl, methylene and aliphatic H atoms at *U*_iso_(H) = 1.2*U*_eq_(C).

### Crystal data

**Table:**

C_23_H_27_NO_3_	*F*(000) = 784
*M**_r_* = 365.46	*D*_x_ = 1.263 Mg m^−^^3^
Monoclinic, *P*2_1_/*n*	Mo *K*α radiation, λ = 0.71073 Å
Hall symbol: -P 2yn	Cell parameters from 4178 reflections
*a* = 7.9569 (3) Å	θ = 2.6–28.0°
*b* = 20.8291 (9) Å	µ = 0.08 mm^−^^1^
*c* = 11.6708 (6) Å	*T* = 298 K
β = 96.297 (2)°	Block, colourless
*V* = 1922.59 (15) Å^3^	0.41 × 0.24 × 0.20 mm
*Z* = 4	

### Data collection

**Table:**

Bruker APEXII CCD area-detector diffractometer	4608 independent reflections
Radiation source: fine-focus sealed tube	3166 reflections with *I* > 2σ(*I*)
graphite	*R*_int_ = 0.026
φ and ω scans	θ_max_ = 28.3°, θ_min_ = 2.6°
Absorption correction: multi-scan (*SADABS*; Bruker, 1999)	*h* = −8→10
*T*_min_ = 0.288, *T*_max_ = 0.980	*k* = −27→27
14049 measured reflections	*l* = −15→15

### Refinement

**Table:**

Refinement on *F*^2^	Primary atom site location: structure-invariant direct methods
Least-squares matrix: full	Secondary atom site location: difference Fourier map
*R*[*F*^2^ > 2σ(*F*^2^)] = 0.048	Hydrogen site location: inferred from neighbouring sites
*wR*(*F*^2^) = 0.127	H atoms treated by a mixture of independent and constrained refinement
*S* = 1.02	*w* = 1/[σ^2^(*F*_o_^2^) + (0.0542*P*)^2^ + 0.4061*P*] where *P* = (*F*_o_^2^ + 2*F*_c_^2^)/3
4608 reflections	(Δ/σ)_max_ < 0.001
251 parameters	Δρ_max_ = 0.22 e Å^−^^3^
0 restraints	Δρ_min_ = −0.21 e Å^−^^3^

### Special details

**Table:**

Geometry. All e.s.d.'s (except the e.s.d. in the dihedral angle between two l.s. planes)are estimated using the full covariance matrix. The cell e.s.d.'s are takeninto account individually in the estimation of e.s.d.'s in distances, anglesand torsion angles; correlations between e.s.d.'s in cell parameters are onlyused when they are defined by crystal symmetry. An approximate (isotropic)treatment of cell e.s.d.'s is used for estimating e.s.d.'s involving l.s. planes.
Refinement. Refinement of *F*^2^ against ALL reflections. The weighted *R*-factor *wR* andgoodness of fit *S* are based on *F*^2^, conventional *R*-factors *R* are basedon *F*, with *F* set to zero for negative *F*^2^. The threshold expression of*F*^2^ > σ(*F*^2^) is used only for calculating *R*-factors(gt) *etc*. and isnot relevant to the choice of reflections for refinement. *R*-factors basedon *F*^2^ are statistically about twice as large as those based on *F*, and *R*-factors based on ALL data will be even larger.

### Fractional atomic coordinates and isotropic or equivalent isotropic displacement parameters (Å^2^)

**Table:**

	*x*	*y*	*z*	*U*_iso_*/*U*_eq_	
C1	0.23693 (17)	0.12454 (6)	0.28186 (12)	0.0323 (3)	
H1	0.1669	0.1269	0.3458	0.039*	
C2	0.11755 (18)	0.10977 (7)	0.16904 (12)	0.0363 (3)	
C3	0.2099 (2)	0.10169 (8)	0.06003 (13)	0.0431 (4)	
H3A	0.1254	0.0972	−0.0060	0.052*	
H3B	0.2725	0.1408	0.0492	0.052*	
C4	0.3315 (2)	0.04531 (8)	0.06032 (14)	0.0473 (4)	
H4A	0.4347	0.0557	0.1089	0.057*	
H4B	0.3604	0.0385	−0.0174	0.057*	
C5	0.2575 (2)	−0.01655 (8)	0.10382 (14)	0.0465 (4)	
H5A	0.3483	−0.0473	0.1211	0.056*	
H5B	0.1790	−0.0345	0.0427	0.056*	
C6	0.16498 (18)	−0.00768 (7)	0.21184 (13)	0.0372 (3)	
H6	0.1050	−0.0475	0.2256	0.045*	
C7	0.27979 (17)	0.01007 (6)	0.32297 (12)	0.0321 (3)	
H7	0.2089	0.0140	0.3862	0.039*	
C8	0.03777 (19)	0.04528 (7)	0.19078 (12)	0.0383 (3)	
C9	0.33357 (18)	0.18729 (6)	0.27865 (12)	0.0334 (3)	
C10	0.49093 (19)	0.18943 (7)	0.23764 (14)	0.0407 (4)	
H10	0.5344	0.1522	0.2082	0.049*	
C11	0.5850 (2)	0.24549 (8)	0.23944 (15)	0.0479 (4)	
H11	0.6903	0.2457	0.2119	0.058*	
C12	0.5214 (2)	0.30076 (8)	0.28224 (16)	0.0505 (4)	
H12	0.5839	0.3385	0.2836	0.061*	
C13	0.3655 (2)	0.30062 (7)	0.32316 (14)	0.0452 (4)	
H13	0.3230	0.3383	0.3516	0.054*	
C14	0.27187 (19)	0.24449 (7)	0.32211 (12)	0.0370 (3)	
C15	−0.0179 (2)	0.16144 (8)	0.14796 (16)	0.0530 (4)	
H15A	−0.0981	0.1490	0.0843	0.079*	
H15B	0.0340	0.2014	0.1305	0.079*	
H15C	−0.0748	0.1665	0.2158	0.079*	
C16	0.41183 (18)	−0.04090 (6)	0.35486 (12)	0.0325 (3)	
C17	0.36550 (18)	−0.09743 (7)	0.40848 (12)	0.0355 (3)	
C18	0.4828 (2)	−0.14549 (7)	0.43706 (13)	0.0432 (4)	
H18	0.4506	−0.1831	0.4717	0.052*	
C19	0.6482 (2)	−0.13728 (8)	0.41378 (14)	0.0487 (4)	
H19	0.7271	−0.1695	0.4330	0.058*	
C20	0.6970 (2)	−0.08216 (8)	0.36268 (15)	0.0497 (4)	
H20	0.8087	−0.0767	0.3481	0.060*	
C21	0.57849 (19)	−0.03438 (7)	0.33286 (14)	0.0421 (4)	
H21	0.6118	0.0028	0.2974	0.050*	
C22	0.0402 (2)	0.29848 (8)	0.39536 (17)	0.0566 (5)	
H22A	0.1056	0.3174	0.4608	0.085*	
H22B	−0.0717	0.2894	0.4143	0.085*	
H22C	0.0339	0.3278	0.3316	0.085*	
C23	0.1527 (3)	−0.15186 (11)	0.49954 (19)	0.0793 (7)	
H23A	0.1658	−0.1920	0.4610	0.119*	
H23B	0.0368	−0.1466	0.5134	0.119*	
H23C	0.2235	−0.1515	0.5717	0.119*	
N1	0.35903 (15)	0.07229 (5)	0.30669 (11)	0.0324 (3)	
O1	−0.11245 (14)	0.03744 (6)	0.19326 (12)	0.0606 (4)	
O2	0.11835 (14)	0.24067 (5)	0.36491 (10)	0.0498 (3)	
O3	0.19994 (13)	−0.10095 (5)	0.42960 (10)	0.0484 (3)	
H1A	0.4203 (18)	0.0824 (7)	0.3696 (13)	0.032 (4)*	

### Atomic displacement parameters (Å^2^)

**Table:**

	*U*^11^	*U*^22^	*U*^33^	*U*^12^	*U*^13^	*U*^23^
C1	0.0345 (7)	0.0274 (7)	0.0357 (7)	0.0025 (5)	0.0074 (6)	0.0021 (6)
C2	0.0341 (8)	0.0356 (8)	0.0387 (8)	0.0037 (6)	0.0025 (6)	0.0037 (6)
C3	0.0492 (9)	0.0446 (9)	0.0360 (8)	−0.0024 (7)	0.0065 (7)	0.0062 (7)
C4	0.0524 (10)	0.0531 (10)	0.0386 (8)	0.0012 (8)	0.0149 (7)	−0.0044 (7)
C5	0.0556 (10)	0.0417 (9)	0.0415 (9)	0.0027 (7)	0.0019 (7)	−0.0094 (7)
C6	0.0371 (8)	0.0303 (7)	0.0437 (8)	−0.0074 (6)	0.0027 (6)	−0.0005 (6)
C7	0.0336 (7)	0.0281 (7)	0.0356 (7)	−0.0004 (6)	0.0079 (6)	0.0008 (6)
C8	0.0346 (8)	0.0454 (9)	0.0344 (7)	−0.0040 (7)	0.0021 (6)	0.0012 (6)
C9	0.0375 (8)	0.0278 (7)	0.0352 (7)	0.0011 (6)	0.0047 (6)	0.0033 (6)
C10	0.0406 (8)	0.0338 (8)	0.0490 (9)	0.0025 (6)	0.0104 (7)	0.0025 (7)
C11	0.0397 (9)	0.0431 (9)	0.0625 (11)	−0.0038 (7)	0.0124 (8)	0.0071 (8)
C12	0.0538 (10)	0.0348 (9)	0.0637 (11)	−0.0107 (7)	0.0096 (8)	0.0045 (8)
C13	0.0571 (10)	0.0285 (8)	0.0511 (9)	0.0001 (7)	0.0099 (8)	−0.0009 (7)
C14	0.0426 (8)	0.0314 (7)	0.0379 (8)	0.0021 (6)	0.0085 (6)	0.0037 (6)
C15	0.0466 (10)	0.0492 (10)	0.0613 (11)	0.0119 (8)	−0.0018 (8)	0.0049 (8)
C16	0.0365 (8)	0.0272 (7)	0.0340 (7)	0.0002 (6)	0.0053 (6)	−0.0024 (6)
C17	0.0404 (8)	0.0320 (7)	0.0337 (7)	−0.0031 (6)	0.0025 (6)	−0.0014 (6)
C18	0.0578 (10)	0.0298 (7)	0.0408 (8)	0.0021 (7)	0.0006 (7)	0.0024 (6)
C19	0.0536 (10)	0.0414 (9)	0.0498 (9)	0.0186 (8)	−0.0003 (8)	−0.0031 (7)
C20	0.0395 (9)	0.0502 (10)	0.0608 (10)	0.0094 (7)	0.0116 (8)	−0.0031 (8)
C21	0.0401 (8)	0.0365 (8)	0.0510 (9)	0.0002 (7)	0.0117 (7)	0.0023 (7)
C22	0.0599 (11)	0.0430 (10)	0.0702 (12)	0.0106 (8)	0.0220 (9)	−0.0069 (8)
C23	0.0611 (13)	0.0974 (16)	0.0793 (14)	−0.0177 (11)	0.0073 (11)	0.0504 (13)
N1	0.0324 (6)	0.0256 (6)	0.0383 (7)	−0.0002 (5)	−0.0004 (5)	−0.0006 (5)
O1	0.0336 (6)	0.0681 (8)	0.0795 (9)	−0.0079 (6)	0.0039 (6)	0.0119 (7)
O2	0.0552 (7)	0.0316 (6)	0.0678 (8)	0.0036 (5)	0.0292 (6)	−0.0024 (5)
O3	0.0429 (6)	0.0469 (7)	0.0564 (7)	−0.0067 (5)	0.0093 (5)	0.0167 (5)

### Geometric parameters (Å, °)

**Table:**

C1—N1	1.4663 (17)	C12—C13	1.377 (2)
C1—C9	1.5191 (19)	C12—H12	0.9300
C1—C2	1.5672 (19)	C13—C14	1.386 (2)
C1—H1	0.9800	C13—H13	0.9300
C2—C8	1.519 (2)	C14—O2	1.3719 (17)
C2—C15	1.524 (2)	C15—H15A	0.9600
C2—C3	1.547 (2)	C15—H15B	0.9600
C3—C4	1.521 (2)	C15—H15C	0.9600
C3—H3A	0.9700	C16—C21	1.385 (2)
C3—H3B	0.9700	C16—C17	1.4016 (19)
C4—C5	1.526 (2)	C17—O3	1.3685 (17)
C4—H4A	0.9700	C17—C18	1.384 (2)
C4—H4B	0.9700	C18—C19	1.384 (2)
C5—C6	1.539 (2)	C18—H18	0.9300
C5—H5A	0.9700	C19—C20	1.369 (2)
C5—H5B	0.9700	C19—H19	0.9300
C6—C8	1.499 (2)	C20—C21	1.389 (2)
C6—C7	1.547 (2)	C20—H20	0.9300
C6—H6	0.9800	C21—H21	0.9300
C7—N1	1.4628 (17)	C22—O2	1.4181 (18)
C7—C16	1.5111 (19)	C22—H22A	0.9600
C7—H7	0.9800	C22—H22B	0.9600
C8—O1	1.2099 (18)	C22—H22C	0.9600
C9—C10	1.389 (2)	C23—O3	1.414 (2)
C9—C14	1.4044 (19)	C23—H23A	0.9600
C10—C11	1.386 (2)	C23—H23B	0.9600
C10—H10	0.9300	C23—H23C	0.9600
C11—C12	1.373 (2)	N1—H1A	0.862 (15)
C11—H11	0.9300		
			
N1—C1—C9	108.50 (11)	C12—C11—H11	120.3
N1—C1—C2	110.35 (11)	C10—C11—H11	120.3
C9—C1—C2	114.20 (11)	C11—C12—C13	120.41 (15)
N1—C1—H1	107.9	C11—C12—H12	119.8
C9—C1—H1	107.9	C13—C12—H12	119.8
C2—C1—H1	107.9	C12—C13—C14	120.17 (15)
C8—C2—C15	110.51 (13)	C12—C13—H13	119.9
C8—C2—C3	106.63 (12)	C14—C13—H13	119.9
C15—C2—C3	109.61 (13)	O2—C14—C13	123.04 (13)
C8—C2—C1	105.02 (11)	O2—C14—C9	116.28 (12)
C15—C2—C1	110.47 (12)	C13—C14—C9	120.67 (14)
C3—C2—C1	114.42 (12)	C2—C15—H15A	109.5
C4—C3—C2	116.20 (12)	C2—C15—H15B	109.5
C4—C3—H3A	108.2	H15A—C15—H15B	109.5
C2—C3—H3A	108.2	C2—C15—H15C	109.5
C4—C3—H3B	108.2	H15A—C15—H15C	109.5
C2—C3—H3B	108.2	H15B—C15—H15C	109.5
H3A—C3—H3B	107.4	C21—C16—C17	117.99 (13)
C3—C4—C5	112.60 (14)	C21—C16—C7	122.65 (12)
C3—C4—H4A	109.1	C17—C16—C7	119.37 (13)
C5—C4—H4A	109.1	O3—C17—C18	123.70 (13)
C3—C4—H4B	109.1	O3—C17—C16	115.52 (12)
C5—C4—H4B	109.1	C18—C17—C16	120.78 (14)
H4A—C4—H4B	107.8	C19—C18—C17	119.62 (14)
C4—C5—C6	114.05 (12)	C19—C18—H18	120.2
C4—C5—H5A	108.7	C17—C18—H18	120.2
C6—C5—H5A	108.7	C20—C19—C18	120.67 (14)
C4—C5—H5B	108.7	C20—C19—H19	119.7
C6—C5—H5B	108.7	C18—C19—H19	119.7
H5A—C5—H5B	107.6	C19—C20—C21	119.53 (16)
C8—C6—C5	109.21 (12)	C19—C20—H20	120.2
C8—C6—C7	106.72 (11)	C21—C20—H20	120.2
C5—C6—C7	115.07 (12)	C16—C21—C20	121.40 (14)
C8—C6—H6	108.6	C16—C21—H21	119.3
C5—C6—H6	108.6	C20—C21—H21	119.3
C7—C6—H6	108.6	O2—C22—H22A	109.5
N1—C7—C16	110.89 (11)	O2—C22—H22B	109.5
N1—C7—C6	109.03 (11)	H22A—C22—H22B	109.5
C16—C7—C6	111.68 (11)	O2—C22—H22C	109.5
N1—C7—H7	108.4	H22A—C22—H22C	109.5
C16—C7—H7	108.4	H22B—C22—H22C	109.5
C6—C7—H7	108.4	O3—C23—H23A	109.5
O1—C8—C6	123.22 (14)	O3—C23—H23B	109.5
O1—C8—C2	123.74 (14)	H23A—C23—H23B	109.5
C6—C8—C2	113.02 (12)	O3—C23—H23C	109.5
C10—C9—C14	117.47 (13)	H23A—C23—H23C	109.5
C10—C9—C1	120.91 (12)	H23B—C23—H23C	109.5
C14—C9—C1	121.54 (13)	C7—N1—C1	113.43 (11)
C11—C10—C9	121.82 (14)	C7—N1—H1A	108.5 (10)
C11—C10—H10	119.1	C1—N1—H1A	106.7 (10)
C9—C10—H10	119.1	C14—O2—C22	118.30 (12)
C12—C11—C10	119.47 (15)	C17—O3—C23	117.79 (13)
			
N1—C1—C2—C8	56.53 (14)	C9—C10—C11—C12	−0.4 (3)
C9—C1—C2—C8	179.06 (12)	C10—C11—C12—C13	0.0 (3)
N1—C1—C2—C15	175.70 (12)	C11—C12—C13—C14	0.4 (3)
C9—C1—C2—C15	−61.77 (16)	C12—C13—C14—O2	177.88 (14)
N1—C1—C2—C3	−60.04 (15)	C12—C13—C14—C9	−0.5 (2)
C9—C1—C2—C3	62.49 (16)	C10—C9—C14—O2	−178.29 (13)
C8—C2—C3—C4	−51.75 (17)	C1—C9—C14—O2	−1.4 (2)
C15—C2—C3—C4	−171.39 (14)	C10—C9—C14—C13	0.2 (2)
C1—C2—C3—C4	63.89 (17)	C1—C9—C14—C13	177.06 (13)
C2—C3—C4—C5	44.90 (19)	N1—C7—C16—C21	−20.15 (19)
C3—C4—C5—C6	−43.79 (19)	C6—C7—C16—C21	101.66 (15)
C4—C5—C6—C8	51.92 (17)	N1—C7—C16—C17	160.12 (12)
C4—C5—C6—C7	−68.05 (17)	C6—C7—C16—C17	−78.06 (16)
C8—C6—C7—N1	−58.41 (14)	C21—C16—C17—O3	179.13 (12)
C5—C6—C7—N1	62.92 (15)	C7—C16—C17—O3	−1.14 (19)
C8—C6—C7—C16	178.70 (11)	C21—C16—C17—C18	−0.8 (2)
C5—C6—C7—C16	−59.97 (16)	C7—C16—C17—C18	178.93 (13)
C5—C6—C8—O1	119.23 (16)	O3—C17—C18—C19	−179.09 (14)
C7—C6—C8—O1	−115.79 (16)	C16—C17—C18—C19	0.8 (2)
C5—C6—C8—C2	−62.18 (15)	C17—C18—C19—C20	−0.1 (2)
C7—C6—C8—C2	62.80 (15)	C18—C19—C20—C21	−0.7 (3)
C15—C2—C8—O1	−1.6 (2)	C17—C16—C21—C20	0.0 (2)
C3—C2—C8—O1	−120.63 (16)	C7—C16—C21—C20	−179.71 (14)
C1—C2—C8—O1	117.57 (16)	C19—C20—C21—C16	0.7 (3)
C15—C2—C8—C6	179.84 (13)	C16—C7—N1—C1	−176.79 (11)
C3—C2—C8—C6	60.79 (15)	C6—C7—N1—C1	59.86 (15)
C1—C2—C8—C6	−61.01 (15)	C9—C1—N1—C7	174.42 (11)
N1—C1—C9—C10	34.09 (17)	C2—C1—N1—C7	−59.77 (15)
C2—C1—C9—C10	−89.44 (16)	C13—C14—O2—C22	9.9 (2)
N1—C1—C9—C14	−142.65 (13)	C9—C14—O2—C22	−171.66 (14)
C2—C1—C9—C14	93.82 (16)	C18—C17—O3—C23	9.8 (2)
C14—C9—C10—C11	0.2 (2)	C16—C17—O3—C23	−170.10 (16)
C1—C9—C10—C11	−176.64 (14)		

### Hydrogen-bond geometry (Å, °)

**Table:**

*D*—H···*A*	*D*—H	H···*A*	*D*···*A*	*D*—H···*A*
N1—H1A···Cg1^i^	0.862 (15)	2.852 (3)	3.6276 (14)	150.6 (12)

Symmetry codes: (i) −*x*+1, −*y*, −*z*+1.
